# QTc prolongation in adolescents with acute alcohol intoxication

**DOI:** 10.1007/s00431-022-04471-2

**Published:** 2022-04-28

**Authors:** Loes de Veld, Nico van der Lely, Ben J. M. Hermans, Joris J. van Hoof, Lichelle Wong, Arja Suzanne Vink

**Affiliations:** 1 Erasmus School of Health Policy and Management, Erasmus University, Postbus 1738, 3000 Rotterdam, DR Netherlands; 2 Department of Pediatrics, Reinier de Graaf Hospital, Delft, Netherlands; 3 Faculty of Medicine and Health Sciences, University of Antwerp, Antwerp, Belgium; 4 Department of Physiology, Cardiovascular Research Institute Maastricht (CARIM), Maastricht University, Maastricht, Netherlands; 5 Department of Cardiology, Heart Center, Amsterdam UMC, University of Amsterdam, Amsterdam, Netherlands; 6 Department of Pediatric Cardiology, Emma Children’s Hospital, Amsterdam UMC, University of Amsterdam, Amsterdam, Netherlands

**Keywords:** Adolescents, Alcohol intoxication, Electrocardiogram, QTc prolongation

## Abstract

In adults, alcohol intoxication is associated with prolongation of the QT interval corrected for heart rate (QTc). The QTc is influenced by age and sex. Although alcohol intoxication is increasingly common in adolescents, there are no data on the prevalence of QTc prolongation in adolescents with alcohol intoxication. This study aimed to determine the prevalence of QTc prolongation in adolescents with alcohol intoxication and identify at-risk adolescents. In this observational study including adolescents aged 10–18 years, heart rate and QT interval were automatically assessed from an electrocardiogram (ECG) at alcohol intoxication using a validated algorithm. The QTc was calculated using both the Bazett formula (QTc_B_) and Fridericia formula (QTc_F_). If present, an ECG recorded within 1 year of the date of admission to the emergency department was obtained as a reference ECG. A total of 317 adolescents were included; 13.3% had a QTc_B_ and 7.9% a QTc_F_ longer than the sex- and age-specific 95th-percentile. None of the adolescents had a QTc_B_ or QTc_F_ > 500 ms, but 11.8% of the adolescents with a reference ECG had a QTc_B_ prolongation of > 60 ms, while no adolescents had a QTc_F_ prolongation of > 60 ms. QTc prolongation was mainly attributable to an increase in heart rate rather than QT prolongation, which underlies the differences between QTc_B_ and QTc_F_. Male sex and hypokalaemia increased the likelihood of QTc prolongation.

*Conclusion*: QTc prolongation was seen in approximately 10% of the adolescents presenting with alcohol intoxication, and although no ventricular arrhythmias were observed in this cohort, QTc prolongation increases the potential for malignant QT-related arrhythmias. Clinicians must be aware of the possibility of QTc prolongation during alcohol intoxication and make an effort to obtain an ECG at presentation, measure the QT interval, and give an adequate assessment of the findings. We advocate admitting adolescents with alcohol intoxication and QTc prolongation. During hospital admission, we recommend limiting exposure to QTc-prolonging medication, increasing potassium levels to a high-normal range (4.5–5.0 mmol/L) and obtaining a reference ECG at discharge.

**Supplementary information:**

The online version contains supplementary material available at 10.1007/s00431-022-04471-2.

**Table:** 

**What is Known:** *• One out of five deaths in adolescents is alcohol-related. Alcohol intoxication has been related to cardiac arrhythmias and sudden cardiac death*. *•In adults, alcohol intoxication is associated with QTc prolongation*.	
**What is New:** *•*Approximately 10% of the adolescents with alcohol intoxication had a QTc longer than the age- and sexspecific cut-off. In contrast to adults, in adolescents with alcohol intoxication, QTc prolongation is attributable to an increase in heart rate, rather than a prolongation of the QT interval. *•Especially males and adolescents with hypokalaemia are at risk of QTc prolongation*.	

## Introduction

Alcohol is the most commonly used psychoactive substance among adolescents [1, 2] and can lead to major alcohol-attributed health risks and even death [3]. In recent decades, alcohol intoxication has become an increasing problem in adolescents with rising admissions to the emergency department and rates of hospitalization [4–8]. One out of five deaths in adolescents is even related to alcohol, with approximately 5% being due to cardiovascular causes [3]. Alcohol intoxication is associated with cardiac arrhythmias and sudden cardiac death [9–17].

Cardiovascular symptoms, such as tachycardia and hypotension, caused by both volume depletion (due to inhibition of antidiuretic hormone and vomiting) and vasodilatation have been reported in adolescents with alcohol intoxication [18, 19]. As alcohol intoxication can induce biochemical changes, such as hypoglycaemia and electrolyte disturbances (such as hypokalaemia, hypernatremia, and hyperchloremia) [19–21], there is a potential risk of cardiac arrhythmias. Guidelines advocate performing an ECG when there is evidence of illicit drug use [22] but do not have specific recommendations for alcohol intoxication. However, in clinical practice in adults, an ECG is obtained in most cases [23]. From that, we know that alcohol intoxication is associated with ECG changes, most frequently prolongation of the QT interval corrected for heart rate (QTc) [23–25]. QTc prolongation predisposes the patient to a life-threatening ventricular arrhythmia, known as Torsade de Pointes (TdP) [26] that can precipitate syncope, sudden cardiac arrest, or sudden cardiac death [26]. There are currently, however, no data on the prevalence of QTc prolongation in adolescents with alcohol intoxication.

The QTc is influenced by age and sex, probably under the influence of sex hormones [27]. Puberty is an important transition period during which changes in the QTc occur, with no sex differences in the QTc before the onset of puberty, but thereafter, a longer QTc is present in females compared to males. In patients with long QT syndrome (LQTS), puberty plays an important role in the sex-related risk for cardiac events [27, 28]. We therefore postulate that individuals in the puberty transition period, i.e., adolescents, are more sensitive to modulators that affect the QTc, such as alcohol intoxication. We therefore aimed to determine the prevalence of QTc prolongation and ventricular arrhythmias in adolescents presenting with alcohol intoxication. Additionally, we wanted to identify adolescents at risk for QTc prolongation.

## Materials and method

### Study design and setting

In this single-centre, retrospective, observational study, we enrolled adolescents aged 10–18 years with a blood alcohol concentration (BAC) > 0.0 g/L who were admitted to the emergency department of the Reinier de Graaf Hospital in Delft, the Netherlands, between January 2009 and December 2019. Adolescents with a history of heart disease were excluded.

### Collection of ECGs and additional data

The first recorded 12-lead ECG during alcohol intoxication was obtained (ECG_intox_) from all of the included adolescents. ECGs that were not available digitally or were recorded in the presence of conduction disorders or pre-excitation were excluded from the analysis. To compare the ECG_intox_ to baseline conditions, an ECG recorded within 1 year before or after the date of admission to the emergency department was obtained (ECG_reference_). All ECGs were digitalized and blinded to patient characteristics.

Additional adolescent characteristics were collected, including age, sex, vital functions, urine toxicology screening results (illicit drug use), electrolyte and serum glucose levels, pH, BAC, and medication usage. QT-prolonging medication was defined as described in CredibleMeds [29].

### ECG measurements

The RR interval and QT interval were automatically assessed using a previously validated algorithm [30]. All annotations were checked manually and edited when necessary. Heart rate (HR) was calculated from the RR interval, and the QTc was calculated using both the Bazett (QTc_B_) [31] and Fridericia (QTc_F_) formulas [32]. Bazett’s formula is the most widely used in clinical practice and for research purposes and therefore enables comparisons to previous studies. However, since Bazett’s formula possibly overcorrects the QT interval at higher heart rates [33] and tachycardia occurs in 10% of children with alcohol intoxication [18], we also calculated the QTc with Fridericia’s formula.

### Data analyses

All data were analyzed using IBM SPSS Statistics version 25.0 for Windows (IBM Corp, Armonk, NY). The ECG measurements and baseline characteristics are presented as numbers (percentage, %) for categorical variables and as the mean (standard deviation, SD, normal distribution) or median (interquartile range, IQR, skewed distribution) for continuous variables. Age- and sex-specific cut-off values for the QTc were based on the 95th percentile: QTc_B_ > 430 ms or QTc_F_ > 420 for males and QTc_B_ > 450 ms or QTc_F_ > 430 ms for females [34]. In addition, the risk for TdP was estimated based on the prevalence of a QTc > 500 ms [35] or a QTc increase > 60 ms between ECG_intox_ and ECG_reference_ [36]. A *p* value < 0.05 was considered to be statistically significant.

To identify the adolescents at risk for QTc prolongation, we performed a two-phase analysis. First, we performed a Pearson’s correlation test for continuous variables and a point-biserial correlation test for dichotomous variables to identify univariate correlations between the QTc and potential predictors for QTc prolongation. This analysis was also performed for HR and the QT interval to gain insight into the effect on the QTc, by either the effect on the HR or the effect on the QT interval. Second, we performed multivariable logistic regression analyses based on statistically significant correlation coefficients and clinical knowledge of confounding factors for QTc prolongation. *P* values were adjusted using the Holm–Bonferroni method due to multiple testing [37].

## Results

### Population characteristics

**Fig. 1:**
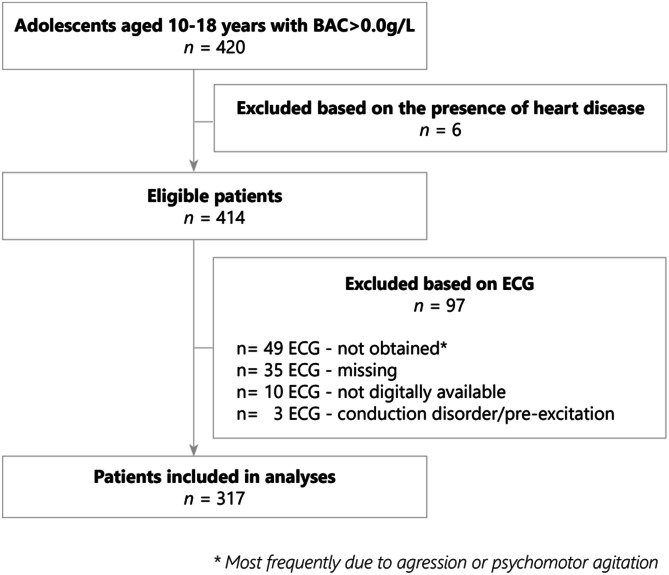
Flowchart of study population

From a total of 420 adolescents who were eligible for the study, 103 (24.5%) were excluded (Fig. 1) due to underlying heart disease (*n* = 6, 5.8%) or on the basis of ECG characteristics (*n* = 97, 23.1%). The remaining 317 adolescents were included in the analysis. Adolescents excluded based on ECG characteristics were hospitalized less frequently than adolescents in whom an ECG_intox_ was available (Supplementary Table S1). None of the adolescents excluded based on ECG characteristics presented with TdP.

The baseline characteristics of the included adolescents are shown in Table 1. The median age was 16 years (IQR 1.0 years), with no patients aged < 12 years and a slight female predominance (57.1%). Most adolescents did not use medication (76.7%); however, 32 (10.1%) used medications associated with QT prolongation, mainly chronic (psychopharmacological) medications, such as methylphenidate. The mean BAC was 1.9 g/L (SD 0.6 g/L), and 31 (9.8%) adolescents had a positive urine toxicology screening. None of the adolescents presented with TdP. A reference ECG was available for 34 (10.7%) adolescents.

**Table 1:** Baseline characteristics

Characteristics		*n* = 317
Demographic characteristics		
Females		181 (57.1%)
Age *in years*		16.0 (IQR 1.0)
Intoxication characteristics		
Medication usage		
* None*		243 (76.7%)
* Medication not associated with QT interval prolongation*		42 (13.2%)
* Medication associated with QT interval prolongation*		32 (10.1%)
BAC *in g/L*		1.9 (SD 0.6)
Illicit drug use		31 (9.8%)
Vital functions and monitoring		
Body temperature *in °C*		36.0 (IQR 1.0)
Glasgow Coma Scale *in EMV score*		14 (IQR 2)
Heart rate *in bmp*		88 (IQR 26)
Systolic blood pressure *in mmHg*		114 (SD 14)
TdP or other ventricular arrhythmias		0 (0.0%)
Follow-up		
Reference ECG		34 (10.7%)
Hospital admission		288 (90.9%)

*BAC* blood alcohol concentration, *bpm* beats per minute, *ECG* electrocardiogram, *EMV* eye response verbal response motor response, *IQR* interquartile range, *n* sample size, *SD* standard deviation, *TdP* Torsade de Pointes. Baseline characteristics are presented as numbers (percentage, %) for categorical variables and as mean (standard deviation, SD, normal distribution) or median (interquartile range, IQR, non-normal distribution) for continuous variables

The laboratory findings of the adolescents with alcohol intoxication are shown in Supplementary Table S2. The most common electrolyte disturbances were hyperchloremia (39.1%), hypokalaemia (23.9%), hypocalcaemia (18.5%), and hypernatremia (7.6%).

### ECG measurements during alcohol intoxication

ECG characteristics stratified by sex are shown in Table 2, including data from 181 females and 136 males. The mean HR was significantly higher in females than in males (93 bpm versus 84 bpm, *p* < 0.001), while there was no statistically significant difference in the QT interval (344 ms versus 346 ms, *p* = 0.52). As a consequence, the QTc was significantly longer in females than in males (QTc_B_ 422 ms versus 404 ms, *p* < 0.001; QTc_F_ 393 ms versus 384 ms, *p* = 0.008). This finding remained present when the data were stratified by age (Supplementary Table S3). The proportion of adolescents with a QTc above the cut-off value did not differ significantly between females and males (QTc_B_ 11.0% versus 16.9%, *p* = 0.13; QTc_F_ 6.1% versus 10.3%, *p* = 0.17). Adolescents using QT-prolonging medication did not show a significant difference in the proportion of participants with QTc prolongation compared to adolescents who did not use QT-prolonging medication (QTc_B_ 13.7% versus 12.5%, *p* = 0.83; QTc_F_ 8.1% versus 6.3%, *p* = 0.72). None of the adolescents had a QTc > 500 ms.

**Table 2:** ECG characteristics of adolescents with alcohol intoxication stratified by sex

**Characteristics**	**ECG**_intox_	
	***Mean (SD)***	***Min–Max***
Heart rate in bpm		
*Females*	93 (18)	48–159
*Males*	84 (18)	49–127
QT interval in msec		
*Females*	344 (35)	251–469
*Males*	346 (35)	275–422
QTc_B_ in msec		
*Females*	422 (22)	367–476
*Males*	404 (30)	321–491
QTc_F_ in msec		
*Females*	394 (21)	340–452
*Males*	383 (26)	326–451

This table shows the ECG characteristics of 181 females and 136 males

*Bpm* beats per minute, *msec* milliseconds, *QTc*_*B*_ QT interval corrected for heart rate by Bazett’s formula, *QTc*_*F*_ QT interval corrected for heart rate by Fridericia’s formula, *SD* standard deviation

### ECG measurements compared to baseline conditions

From the 34 adolescents with a reference ECG, the ECG_reference_ was most often recorded at discharge (76.4%) or within 6 months after emergency department presentation (20.6%). Adolescents with a reference ECG more frequently had a QTc longer than age- and sex-specific cut-off values compared to those who did not have a reference ECG (Supplementary S4). Furthermore, although not statistically significant (*p* = 0.06), adolescents with a reference ECG used QT-prolonging medication more often than those who did not have a reference ECG (20.6% versus 8.8%). However, five out of seven used the medication chronically and during both ECG recordings. One adolescent was on a clarithromycin course on the day of emergency department presentation, and one received one dose of metoclopramide at the emergency department due to profuse vomiting.

**Fig. 2:**
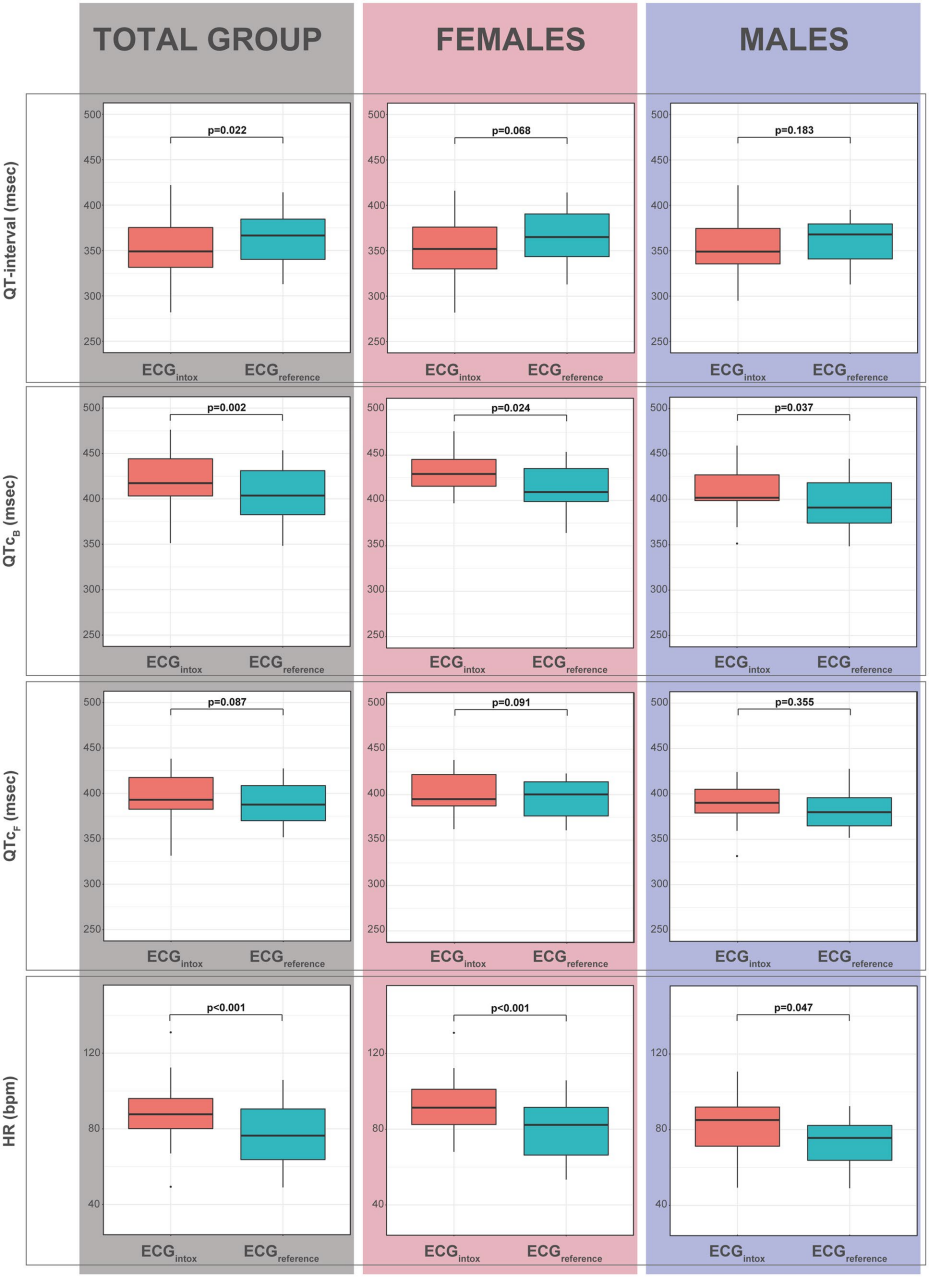
Boxplot QT interval, QTc, and heart rate between ECG_intox_ and ECG_reference_ in 34 adolescents. Note: bpm, beats per minute; ECG, electrocardiogram; HR, heart rate; msec, milliseconds

In Fig. 2, the differences between ECG_intox_ and ECG_reference_ are shown. There was a significantly higher HR at the time of alcohol intoxication compared to the time of ECG_reference_ acquisition (88 bpm versus 76 bpm, *p* < 0.001) and a shorter QT interval (351 ms versus 362 ms, *p* = 0.022). Interestingly, there was a longer QTc_B_ (421 ms versus 405 ms, *p* = 0.002) for ECG_intox_ compared to ECG_reference_, while no significant difference was seen in QTc_F_ (396 ms versus 390 ms, *p* = 0.18). There were no significant sex differences for either QTc_B_ or QTc_F_.

Table 3 shows the extent to which the QTc differs between ECG_intox_ and ECG_reference_. Compared to baseline conditions, 70.6% of the adolescents had a QTc_B_ prolongation of up to 30 ms during alcohol intoxication, whereas this was only 44.1% for QTc_F_. Remarkably, females seemed to have more variability in QTc_B_ between ECG_intox_ and ECG_reference_ than males, which was not evident for QTc_F_. Four adolescents (11.8%) had a QTc_B_ prolongation > 60 ms, while this was not seen for QTc_F_. Of these four adolescents (Table 4), three had a larger HR increase between ECG_intox_ and ECG_reference_ than the mean HR increase (~40 bpm versus 12 bpm). In all four adolescents, there was an additional factor for QTc prolongation, namely, hypokalaemia (*n* = 1), hypocalcaemia (*n* = 2), hypernatremia (*n* = 1), acidosis (*n* = 2), metoclopramide (*n* = 1), and (meth)amphetamine intoxication [38].

**Table 3:** Differences in QTc (ΔQTc) between ECG_intox_ and ECG_reference_ stratified by sex

	**Reference category**	ΔQTc ** − 30–60 ms**	ΔQTc ** − 0–30 ms**	ΔQTc ** + 0–30 ms**	ΔQTc ** + 30–60 ms**	ΔQTc ** > + 60 ms**
**QTc**_**B**_	Females (n = 19)	2 (10.5%)	3 (15.8%)	8 (42.1%)	4 (21.1%)	2 (10.5%)
	Males (n = 15)	0 (0.0%)	5 (33.3%)	7 (46.7%)	1 (6.7%)	2 (13.3%)
	Total (n = 34)	2 (5.9%)	8 (23.5%)	15 (44.1%)	5 (14.7%)	4 (11.8%)
	Females (n = 19)	2 (10.5%)	9 (47.4%)	7 (36.8%)	1 (5.3%)	0 (0.0%)
**QTc**_**F**_	Males (n = 15)	1 (6.7%)	7 (46.7%)	7 (46.7%)	0 (0.0%)	0 (0.0%)
	Total (n = 34)	3 (8.8%)	16 (47.1%)	14 (41.2%)	1 (2.9)	0 (0.0%)

*msec* milliseconds, *n* sample size, *QTc*_*B*_ QT interval corrected for heart rate by Bazett’s formula, *QTc*_*F*_ QT interval corrected for heart rate by Fridericia’s formula

**Table 4:** Characteristics of the four patients presented with a ΔQTc (QTc_intox_ – QTc_reference_) >  + 60 ms

	**Patient 1**	**Patient 2**	**Patient 3**	**Patient 4**
	ΔQTc_B_ + 73 ms	ΔQTc_B_ + 68 ms	ΔQTc_B_ + 65 ms	ΔQTc_B_ + 65 ms
**Demographic characteristics**				
Sex	Girl	Boy	**Boy**	Girl
Age *in years*	16	15	**14**	15
**Intoxication characteristics**				
QT-prolonging medication	-	**Metoclopramide**	-	-
BAC *in g/L*	2.7	2.0	1.4	1.9
Positive urine drug screening	-	**Cannabis**	-	**(Meth)amphetamine**
**Vital functions**				
Body temperature *in °C*	37.5	**35.5**	37.2	**36.0**
Glasgow Coma Score	13	**8**	14	15
Systolic blood pressure *in mmHg*	110	**93**	**140**	100
**Laboratory results**				
Sodium *in mmol/L*	140	**150**	143	142
Potassium *in mmol/L*	**3.0**	4.2	3.8	4.0
Calcium *in mmol/L*	**2.19**	**2.18**	2.25	2.34
Chloride *in mmol/L*	101	**112**	104	**108**
Glucose *in mmol/L*	7.6	6.1	6.0	8.4
Arterial-blood gas *in pH*	-	**7.33**	7.38	**7.29**
**ECG**_**intox**_				
Heart rate *in bpm*	99	**111**	92	70
QT interval *in msec*	371	338	333	398
QTc_B_ *in msec*	**476**	**459**	413	429
QTc_F_ *in msec*	**438**	415	385	419
**ECG**_**reference**_				
Heart rate *in bpm*	60	68	51	53
QT interval *in msec*	403	368	379	386
QTc_B_ *in msec*	403	391	348	364
QTc_F_ *in msec*	403	383	358	371

*BAC* blood alcohol concentration, *bpm* beats per minute, *ECG* electrocardiogram, *msec* milliseconds, *QTc*_*B*_ QT interval corrected for heart rate by Bazett’s formula, *QTc*_*F*_ QT interval corrected for heart rate by Fridericia’s formula. Bold font indicates a value above or below the reference interval

### Predictors of QTc prolongation

Correlation coefficients for the QTc, HR, and QT interval are presented in Supplementary Table S5. In Tables 5 and 6, predictors for QTc prolongation as well as for HR and the QT interval are shown. Males with alcohol intoxication had a 2.74 (95% confidence interval [CI] 1.21–6.23, *p* = 0.02) times higher risk for QTc_B_ prolongation than females, and a 5.31 (95% CI 1.38–20.49, *p* = 0.02) times higher risk for QTc_F_ prolongation. Increasing age was associated with a reduction in the risk for QTc_B_ prolongation (OR 0.59, 95% CI 0.42–0.83, *p* < 0.001); however, this was not seen for QTc_F_ prolongation. Each mmol/L reduction in serum potassium was associated with a 6.41 (95% CI 2.02–20.41, *p* < 0.001) times higher risk for QTc_B_ prolongation and a 32.89 (95% CI 4.71–228.67, *p* < 0.001) high risk for QTc_F_ prolongation. Remarkably, there was no independent effect of BAC or QTc-prolonging medication use.

**Table 5:** Logistic-regression-model of predictors of QTc_B_-prolongation based on age- and sex-specific cut-off values

Predictor	Proportion	Odds ratio	*p* value
**Demographic characteristics**			
Sex			
*Females*	9.0%	REF	REF
*Males*	16.3%	2.70 (1.14–6.39)	*p* = 0.02
Age		0.56 (0.39–0.79)	*p* = 0.001
*12–14*	17.5%		
15–17	10.8%		
**Intoxication characteristics**			
Medication			
*Not associated with QT interval prolongation*	12.1%	REF	REF
*Associated with QT interval prolongation*	12.5%	1.39 (0.40–4.86)	*p* = 0.55
Blood alcohol concentration		0.52 (0.21–1.27)	*p* = 0.15
< 2.0 g/L	13.1%		
≥ 2.0 g/L	11.2%		
**Vital functions**			
Body temperature		0.65 (0.38–1.11)	*p* = 0.11
*Hypothermia* < *35.0*	14.8%		
*Body core temperature* ≥ *35.0*	11.6%		
Glasgow Coma Score		0.92 (0.80–1.07)	*p* = 0.28
*Mild EMV 13–15*	10.3%		
*Moderate EMV 9–12*	20.4%		
*Severe EMV* ≤ *8*	16.7%		
Systolic blood pressure		1.00 (0.97–1.03)	*p* = 0.89
*Hypotension (RRsys* < *100 mmHg)*	14.0%		
*Normotension*	12.1%		
*Hypertension (RRsys* > *130 mmHg)*	11.1%		
**Laboratory parameters**			
Serum sodium		0.97 (0.82–1.16)	*p* = 0.75
< *LLN*	-		
*Within reference interval*	12.2%		
> *ULN*	12.5%		
Serum potassium		0.13 (0.04–0.44)	*p* < 0.001
< *LLN*	19.2%		
*Within reference interval*	10.5%		
> *ULN*	-		
Serum calcium		0.10 (0.00–5.08)	*p* = 0.25
< *LLN*	11.9%		
*Within reference interval*	14.5%		
> *ULN*	-		

Continuous variables were entered in the logistic-regression as such. Categorical variables are also shown in the table for the corresponding proportion of adolescents with a QTc_B_ above the age- and sex-specific cut-off values. A dash indicates that the sample size of that category was ≤ 5 and considered too small to determine the proportion of adolescents with a QTc_B_ above the age- and sex-specific cut-off values. *EMV* eye response motor response verbal response, *LLN* lower limit of normal, *QTc*_*B*_ QT interval corrected for heart rate by Bazett’s formula, *RRsys* systolic blood pressure, *ULN* upper limit of normal, *REF* reference category

**Table 6:** Logistic-regression-model of predictors of QTc_F_-prolongation based on age- and sex-specific cut-off values

Predictor	Proportion	Odds ratio	*p* value
**Demographic characteristics**			
Sex			
*Females*	4.0%	REF	REF
*Males*	9.6%	5.32 (1.38–20.49)	*p* = 0.02
Age		1.39 (0.77–2.48)	*p* = 0.28
*12–14*	1.6%		
15–17	7.6%		
**Intoxication characteristics**			
Medication			
*Not associated with QT interval prolongation*	6.4%	REF	REF
*Associated with QT interval prolongation*	6.3%	0.76 (0.12–4.75)	*p* = 0.77
Blood alcohol concentration		0.49 (0.15–1.64)	*p* = 0.25
< 2.0 g/L	4.8%		
≥ 2.0 g/L	8.3%		
**Vital functions**			
Body temperature		1.02 (0.45–2.31)	*p* = 0.95
*Hypothermia* < *35.0*	11.1%		
*Body core temperature* ≥ *35.0*	5.8%		
Glasgow Coma Score		0.84 (0.69–1.02)	*p* = 0.07
*Mild EMV 13–15*	4.2%		
*Moderate EMV 9–12*	10.2%		
*Severe EMV* ≤ *8*	16.7%		
Systolic blood pressure		0.95 (0.92–0.99)	*p* = 0.02
*Hypotension (RRsys* < *100 mmHg)*	9.3%		
*Normotension*	6.3%		
*Hypertension (RRsys* > *130 mmHg)*	0.0%		
**Laboratory parameters**			
Serum sodium		0.84 (0.65–1.08)	*p* = 0.17
< *LLN*	-		
*Within reference interval*	6.3%		
> *ULN*	8.3%		
Serum potassium		0.03 (0.00–0.21)	*p* < 0.001
< *LLN*	16.4%		
*Within reference interval*	3.5%		
> *ULN*	-		
Serum calcium		0.43 (0.00–100.22)	*p* = 0.76
< *LLN*	8.9%		
*Within reference interval*	6.2%		
> *ULN*	-		

Continuous variables were entered in the logistic-regression as such. Categorical variables were also shown in the table for the corresponding proportion of adolescents with a QTc_B_ above the age- and sex-specific cut-off values. A dash indicates that the sample size of that category was ≤ 5 and considered too small to determine the proportion of adolescents with a QTc_F_ above the age- and sex-specific cut-off values. EMV = Eye response Motor Response Verbal Response, LLN = Lower Limit of Normal, QTc_B_ = QT interval corrected for heart rate by Fridericia’s formula, *RRsys* systolic blood pressure, *ULN* upper limit of normal, *REF* reference category

## Discussion

### Main results

The present study is the first to determine the prevalence of QTc prolongation and TdP in adolescents with alcohol intoxication and to identify patients at risk for QTc prolongation. We found a prevalence of QTc prolongation of approximately 10%. None of the adolescents had a QTc > 500 ms or ventricular arrhythmias. Compared to baseline conditions, most adolescents with alcohol intoxication had a QTc prolongation of up to 30 ms, and only 11.8% had a QTc prolongation of > 60 ms. Risk factors for QTc prolongation were male sex and a lower serum potassium level. A young age, i.e., 12–14 years, was associated with QTc_B_ prolongation but not QTc_F_ prolongation.

### Alcohol intoxication and its effect on HR and QTc prolongation

Experimental studies in healthy adult volunteers administered predetermined doses of alcohol (either ingested or intravenously infused) show a dose–response relationship between the amount of alcohol administered and QTc prolongation [39, 40]. In adults, alcohol levels of 0.4–1.4 g/L are associated with a 10–30 ms prolongation of the QTc, which is mainly attributable to an increase in the QT interval, as HR does not significantly increase after alcohol administration [39, 40]. This phenomenon is also seen in adults presenting to an emergency department for alcohol intoxication [18, 23]. In addition to the findings in adults, we found that most adolescents with alcohol intoxication also had a QTc prolongation of 0–30 ms. However, this was mainly caused by a difference in HR between baseline conditions and the time of alcohol intoxication rather than to an increase in the QT interval.

The more prominent role of HR in adolescents compared to adults can be explained by several mechanisms. First, adolescents have a stronger HR response to environmental changes (e.g., during postural changes, fever, psychosocial stress, and physical exercise [41–45]) than adults due to greater baroreflex sensitivity, which causes a greater autonomic response to either parasympathetic withdrawal [46, 47] or sympathetic stimulation[48].

Second, adolescents reach higher stages of intoxication at a lower BAC [1, 18, 20] than adults. Hence, although BAC is the most objective measure to quantify the level of alcohol intoxication, the extent of alcohol intoxication is influenced by factors such as age, individual body weight, tolerance to alcohol, the percentage of alcohol in the beverage, and the period of alcohol ingestion [18]. In a previous study regarding QTc prolongation in adults with alcohol intoxication, there was a mean BAC of 1.7 g/L, corresponding to the excitement stage of alcohol intoxication characterized by emotional instability and decreased inhibition [24]. In our study, the mean BAC was somewhat similar to that in a study in adults (1.9 g/L). However, as adolescents reach higher stages of alcohol intoxication than adults at a lower BAC, one could postulate that the adolescents in our study were at a more advanced stage of intoxication, the confusion stage. Although there are no available data on HR by intoxication stage, the exaggerated emotions of the confusion stage can be associated with a more substantial HR increase than the excitement stage, as emotions can increase HR compared to baseline [49].

The prominent role of HR in adolescents with alcohol intoxication may underlie the different effects seen in QTc based on the chosen correction formula. Most QT interval correction formulas lead to similar QTc values in the presence of baseline conditions and an HR of approximately 60 bpm [33]. The Bazett formula, however, generally shows a more prominent QTc prolongation than the Fridericia formula when the HR is above 60 bpm [33]. As in our study, the mean HR during alcohol intoxication was 88 bpm (IQR 26 bpm), and this phenomenon could explain the differences found between QTc_B_ and QTc_F_, with a more pronounced QTc_B_ prolongation compared to QTc_F_ prolongation at the time of alcohol intoxication and when compared to a reference ECG. In addition, QTc_B_ was not correlated with body temperature or SBP (parameters associated with HR), which was seen for QTc_F_. As HR decreases with age [50], the younger age group showed an increased risk for QTc_B_ prolongation but not QTc_F_ prolongation.

### Risk factors for QTc prolongation in adolescents with alcohol intoxication

QTc is influenced by age and sex, probably under the influence of sex hormones [27]. Before the onset of puberty, no sex differences in QTc are seen, but thereafter, the QTc shortens in males but not in females [27, 51–53], resulting in a postpubertal QTc that is longer in females than in males. QTc shortening in males after puberty is thought to be caused by testosterone [27, 54]. As our study included individuals with ages corresponding to the pubertal period, i.e., ages 12–18 years, the included males were in a transient period of rising serum testosterone levels [55], and therefore, the QTc-shortening effect of testosterone may not have been fully present, increasing their risk for QTc prolongation in the presence of modulating factors such as alcohol intoxication. The effect of female sex hormones, i.e., oestrogen and progestogen, on the QTc is less clear [27, 54]. In adolescents, female sex hormones are influenced by the menstrual cycle. In healthy adult females, no changes in QTc are seen during the phases of the menstrual cycle, but the HR fluctuates during the menstrual phases [56, 57]. As this also applies to female adolescents, it could be postulated that in the presence of modulating factors such as alcohol intoxication, the HR rather than the QTc will be affected. This could explain why females are more sensitive to HR increase during alcohol intoxication compared to males [58], whereas normally females and males around the age of 16 have a similar HR [50].

Hypokalaemia was not surprisingly associated with QTc prolongation in our study. Low extracellular potassium levels reduce the voltage-gated rapid delayed rectifier outward K + -current, which is critical to phase 3 repolarization of cardiomyocytes and therefore results in prolongation of the QT interval [59]. Hypokalaemia is a common finding in adolescents with alcohol intoxication [13, 14] and results from several mechanisms. First, with acute stress, i.e., hospital admittance and ambulance rides, there is a catecholamine-induced intracellular potassium shift [60]. Second, although less frequently observed than acidosis, alkalosis in patients with alcohol intoxication does occur and might also result in an intracellular potassium shift [59]. Third, vomiting and volume depletion may result in extrarenal or renal potassium loss [60–62].

### Limitations

As our study had a retrospective design, only 88% of the adolescents had ECGs, and 10.7% had a reference ECG. This reflects, however, the daily clinical practice, as there are currently no guidelines regarding recommendations for ECG screening and follow-up. The proportion of those with ECGs made at emergency department presentation due to alcohol intoxication in our study is similar to what is seen in adults [23]. Our follow-up is similar to a previous study in children and adolescents presenting with an overdose/intoxication [63], where it was postulated that follow-up was limited due to (I) a low estimated probability of LQTS, as QT prolongation was attributed to other risk factors (such as hypokalaemia or acidosis), (II) a lapse in communication during the transfer of care, and (III) inadequate recognition of abnormal findings [63, 64].

As the majority of the adolescents did not have a reference ECG available, it is difficult to determine if the prolonged QTc was attributable to intoxication or if it was the patients’ normal QTc. As the absolute prevalence of QTc prolongation was somewhat higher in the adolescents with reference ECGs, this prolongation could have been the motivation for follow-up. Therefore, the result that 12% of the adolescents had a QTc prolongation > 60 ms is most likely an overestimation. However, as age- and sex-specific QTc cut-off values were based on the 95th-percentile of a cohort including LQTS genotype-negative family members [34], it is unlikely that 10% of the adolescents with QTc prolongation during alcohol intoxication had this QTc as their normal QTc.

Although not statistically significant, there was an absolute higher use of QT-prolonging medication in the adolescents with a reference ECG, so the use of QT-prolonging medication could have been the motivation to record an ECG_reference_. The use of QT-prolonging medication results in a reduced repolarization reserve [65], which may result in overestimation of the difference between the QTc at the time of alcohol intoxication and baseline conditions. However, this overestimation would not have affected the main results to a great extent as the number of adolescents with QT-prolonging medication was limited, and we mainly observed a difference in HR between the time of the alcohol intoxication and baseline, rather than an increase in the QT interval.

### Recommendations

Clinicians involved in the acute care of adolescents with alcohol intoxication should be aware of the possibility of QTc prolongation during this period and should therefore always obtain an ECG at presentation and accurately assess the QT interval [66, 67]. Although no ventricular arrhythmias were observed in this cohort, QTc prolongation can predispose patients to malignant QT-related arrhythmias. We advocate admitting adolescents with a QTc longer than the age- and sex-specific cut-off values and if there was an increase of at least 60 ms compared with baseline values, especially in young males and in the presence of hypokalaemia. For continuous cardiac monitoring, general precautions apply, including monitoring for a QTc > 500 ms or a QTc prolongation > 60 ms compared to a baseline ECG. In all these patients, additional awareness should be given to limiting exposure to QTc-prolonging medication and considering increasing potassium levels to a high-normal range (4.5–5.0 mmol/L). A reference ECG should be made at discharge.

## Conclusion

QTc prolongation was seen in approximately 10% of the adolescents presenting with alcohol intoxication, and although no ventricular arrhythmias were observed in this cohort, these patients may be predisposed to malignant QT-related arrhythmias. In particular, young males and adolescents with hypokalaemia are at risk for QTc prolongation. Clinicians must be aware of the possibility of QTc prolongation during alcohol intoxication and make an effort to obtain an ECG at presentation, measure the QT interval, and give an adequate assessment of the findings. We advocate admitting adolescents with alcohol intoxication and QTc prolongation. During hospital admission, we recommend limiting exposure to QTc-prolonging medication, increasing potassium levels to a high-normal range (4.5–5.0 mmol/L) and obtaining a reference ECG at discharge.

## Supplementary information

Below is the link to the electronic supplementary material.

Supplementary file1 (DOCX 36 KB)

## Data Availability

Data can be made available upon request.

## Abbreviations

ANCOVA: Analysis of covariance
ANOVA: Analysis of variance
bpm: Beats per minute
BAC: Blood alcohol concentration
NSCK: Nederlands Signaleringscentrum Kindergeneeskunde (Dutch Pediatric Surveillance Unit)
ECG: Electrocardiogram
F: Females
HR: Heart rate
IQR: Interquartile range
LLN: Lower limit of normal
LQTS: Long-long QT-syndrome
M: Males
msec: Milliseconds
n: Sample size
NA: Not applicable
QTc: QT interval corrected for heart rate
QTc_B_: QT interval corrected for heart rate using Bazett’s correction method
QTc_F_: QT interval corrected for heart rate using Fridericia’s correction method
SD: Standard deviation
SBP: Systolic blood pressure
TdP: Torsade de Pointes
ULN: Upper limit of normal
