# Supplementary material for: QTc prolongation in adolescents with acute alcohol intoxication
Source: Eur J Pediatr. 2022 Apr 28;181(7):2757–70. doi: 10.1007/s00431-022-04471-2 (PMC9192465; doi:10.1007/s00431-022-04471-2)
Supplement: Supplementary file 1 — Supplementary file1 (DOCX 36 KB) [file 431_2022_4471_MOESM1_ESM.docx]

| **Supplementary Table S1** Adolescents with ECG_intox_ versus excluded cases based on ECG | | | | |  |
| --- | --- | --- | --- | --- | --- |
| **Characteristics** | | **Excluded based on ECG**  **n=97** | **ECG_intox_**  **n=317** | **p value** |  |
| **Demographic characteristics** | |  |  |  |  |
| Females | | 55 (56.7%) | 181 (57.1%) | 0.95^a^ |  |
| Age *in years* | | 15.0 (IQR 2.0) | 16.0 (IQR 1.0) | 0.15^c^ |  |
|  | |  |  |  |  |
| **Intoxication characteristics** | |  |  |  |  |
| Medication usage  *None*  *Medication not associated with QT interval prolongation*  *Medication associated with QT interval prolongation* | | *78 (80.4%)*  *7 (7.2%)*  *12 (12.4%)* | *243 (76.7%)*  *42 (13.2%*  *32 (10.1%)* | 0.25^a^ |  |
| BAC *in* *g/L* | | 2.0 (SD 0.6) | 1.9 (SD 0.6) | 0.54^c^ |  |
| Illicit drug use | | 12 (12.4%) | 32 (10.1%) | 0.52^a^ |  |
|  |  |  |  |  |  |
| **Vital functions and monitoring** |  |  |  |  |  |
| Glasgow Coma Scale *in EMV score* |  | 13.0 (SD 2.6) | 13.3 (SD 2.5) | 0.43^c^ |  |
| Body temperature *in °C* |  | 35.9 (SD 0.8) | 36.0 (SD 0.8) | 0.36^c^ |  |
| Systolic blood pressure *in mmHg* |  | 115 (SD 14) | 114 (SD 15) | 0.42^c^ |  |
|  |  |  |  |  |  |
| **Follow-up** |  |  |  |  |  |
| Hospital admission |  | 80 (82.5%) | 288 (91.0%) | 0.02^a^ |  |
|  |  |  |  |  |  |
| **Laboratory results** |  |  |  |  |  |
| Hypernatremia |  | 8 (8.9%) | 24 (7.6%) | 0.69^a^ |  |
| Hypokalaemia |  | 20 (22.5%) | 74 (23.9%) | 0.78^a^ |  |
| Hypocalcaemia |  | 16 (19.3%) | 56 (18.5%) | 0.87^a^ |  |
| Hyperchloremia |  | 36 (41.9%) | 119 (39.1%) | 0.65^a^ |  |
| Glucose |  | 6.3 (SD 1.1) | 6.6 (SD 1.4) | 0.10^c^ |  |
| Acidosis |  | 23 (37.1%) | 107 (39.1%) | 0.78 ^a^ |  |

Note: adolescents were excluded because (I) no ECG could be obtained due to aggression or psychomotor agitation), (I) the ECG was missing, (III) the ECG not digitally available, or (IV) because there were conduction disorders or pre-excitation that influences QT interval measurements. BAC = Blood Alcohol Concentration, bpm = beats per minute, ECG = Electrocardiogram, EMV = Eye response Movement response Verbal response, IQR = interquartile Range. a = Chi-square test, b = Mann-Whitney U-test, c ANCOVA, mean adjusted for age- and sex

| **Supplementary Table S2** Laboratory findings of adolescents presented with alcohol intoxication | | | | | | |
| --- | --- | --- | --- | --- | --- | --- |
|  | **Reference range** | **n** | **Mean (SD)** | **Min - Max** | **<LLN,**  **n (percentage)** | **>ULN**  **n (percentage)** |
| Glucose | 3.5-11.1 mmol/L | 314 | 6.6(SD 1.3) | 3.8-16.1 | 0 (0.0%) | 2 (0.6%) |
| Sodium | 134-145 mmol/L | 316 | 142(SD 2) | 137-150 | 0 (0.0%) | 24 (7.6%) |
| Potassium | 3.5-5.0 mmol/L | 310 | 3.8(SD 0.4) | 2.8-5.6 | 74 (23.9%) | 4 (1.3%) |
| Calcium | 2.20-2.65 mmol/L | 303 | 2.29(SD 0.11) | 2.03-2.63 | 56 (18.5%) | 0 (0.0%) |
| Chloride | 97-107 mmol/L | 304 | 106 (SD 4) | 95-116 | 1 (0.3%) | 119 (39.1%) |
| pH | 7.35-7.45 | 274 | 7.37(SD 0.06) | 7.23-7.64 | 107 (39.1%) | 23 (8.4%) |

Note: BAC = Blood Alcohol Concentration, LLN = Lower Limit of Normal, Max = maximum, Min = minimum, n = sample size, ULN = Upper Limit of Normal, SD = Standard Deviation

| **Supplementary Table S3** Age- and sex- adjusted means QT interval, QTc and HR | | | | |  |
| --- | --- | --- | --- | --- | --- |
| **Predictor** | **QT interval**  ***in msec*** | **QTc_B_**  ***in msec*** | **QTc**_F_  ***in msec*** | **HR**  ***in bpm*** | |
| **Demographic variables** |  |  |  |  | |
| Sex ^a^ | p=0.57 | **p<0.001** | **p<0.001** | **p<0.001** | |
| *Females* | 344 | 421 | 394 | 93 | |
| *Males* | 346 | 404 | 383 | 84 | |
| Age ^b^ | p=0.11 | p=0.37 | p=0.93 | p=0.40 | |
| *12-14* | 335 | 410 | 389 | 92 | |
| 15-17 | 347 | 414 | 389 | 88 | |
|  |  |  |  |  | |
| **Intoxication characteristics** |  |  |  |  | |
| Blood alcohol concentration ^c^ | p=0.34 | p=0.68 | p=0/76 | p=0.69 | |
| <2.0g/L | 345 | 407 | 390 | 86 | |
| ≥2.0g/L | 341 | 405 | 389 | 87 | |
| Medication ^c^ | p=0.95 | **p=0.03** | p=0.56 | p=0.24 | |
| *Medication not associated with QT interval prolongation* | 343 | 401 | 387 | 84 | |
| *Medication associated with QT interval prolongation* | 343 | 411 | 393 | 88 | |
| Illicit drug use^c^ | p=0.06 | p=0.72 | p=0.18 | p=0.08 | |
| *Positive* | 334 | 413 | 384 | 94 | |
| *Negative* | 346 | 414 | 390 | 88 | |
|  |  |  |  |  | |
| **Vital functions** |  |  |  |  | |
| Systolic blood pressure ^c^ | **p=0.004** | p=0.42 | **p=0.002** | **p<0.001** | |
| *Hypotension (RRsys <100mmHg)* | 353 | 409 | 395 | 82 | |
| *Normotension* | 348 | 408 | 390 | 84 | |
| *Hypertension (RRsys>130 mmHg)* | 327 | 402 | 377 | 93 | |
| Glasgow Coma Score ^c^ | **p<0.001** | p=0.48 | p=0.10 | **p=0.01** | |
| *Mild EMV 13-15* | 332 | 407 | 388 | 92 | |
| *Moderate EMV 9-12* | 339 | 403 | 390 | 86 | |
| *Severe EMV≤8* | 358 | 409 | 399 | 80 | |

Note: bpm = beats per minute, EMV = Eye response Motor Response Verbal Response, g/L = gram/Liter, HR = Heart Rate, msec = milliseconds, QTc_B_ = QT interval corrected for heart rate by Bazett’s formula, QTc_F_ = QT interval corrected for heart rate by Fridericia’s formula, RRsys = systolic blood pressure. ^a^ = means corrected for age. ^b^ = means corrected for sex. ^c^ = means corrected for age and sex.

| **Supplementary Table S4** Adolescents with a missing reference ECG versus those with an available reference ECG | | | | |
| --- | --- | --- | --- | --- |
| **Characteristics** | | **Missing**  **reference ECG**  **n=283** | **Available reference ECG**  **n=34** | **p value** |
| **Demographic characteristics** | |  |  |  |
| Females | | 162 (57.2%) | 19 (55.9%) | 0.88^a^ |
| Age *in years* | | 15.0 (IQR 2.0) | 16.0 (IQR 1.0) | 0.88^c^ |
|  | |  |  |  |
| **Intoxication characteristics** | |  |  |  |
| Medication usage  *None*  *Medication not associated with QT interval prolongation*  *Medication associated with QT interval prolongation* | | *218 (77.0%)*  *40 (14.1%)*  *25 (8.8%)* | *25 (73.5%)*  *2 (5.9%)*  *7 (20.6%)* | 0.06^a^ |
| BAC *in* *g/L* | | 1.9 (SD 0.6) | 2.0 (SD 0.5) | 0.65^c^ |
| Illicit drug use | | 29 (10.2%) | 3 (8.8%) | 0.80^a^ |
|  |  |  |  |  |
| **Vital functions and monitoring** |  |  |  |  |
| Glasgow Coma Scale *in EMV points* |  | 13.3 (SD 2.5) | 13.1 (SD 2.6) | 0.62^c^ |
| Body temperature *in °C* |  | 36.0 (SD 0.8) | 35.8 (SD 0.8) | 0.06^c^ |
| Systolic blood pressure *in mmHg* |  | 114 (SD 15) | 110 (SD 15) | 0.15^c^ |
|  |  |  |  |  |
| **Follow-up** |  |  |  |  |
| Hospital admission |  | 256 (90.5%) | 32 (94.1%) | 0.48^a^ |
|  |  |  |  |  |
| **Laboratory results** |  |  |  |  |
| Hypernatremia |  | *22 (7.8%)* | *2 (5.9%)* | 0.69^a^ |
| Hypokalaemia |  | *65 (23.6%)* | *9 (26.5%)* | 0.71^a^ |
| Hypocalcaemia |  | *50 (18.5%)* | *6 (18.2%)* | 0.96^a^ |
| Hyperchloremia |  | 105 (38.7%) | 14 (42.4%) | 0.68^a^ |
| Glucose |  | 6.6 (SD 1.3) | 6.6 (SD 1.4) | 0.91^c^ |
| Acidosis |  | 89 (36.6%) | 31 (58.1%) | **0.02 ^a^** |
|  |  |  |  |  |
| **ECG_intox_ parameters** |  |  |  |  |
| Heart rate *in* *bpm* |  | 89 (SD 18) | 88 (SD 18) | 0.89^c^ |
| QT interval *in* *msec* |  | 344 (SD 35) | 351 (SD 35) | 0.32^c^ |
| QTc_B_ *in msec* |  | 413 (SD 25) | 421 (SD 26) | 0.08^c^ |
| QTC_B_ > 95^th^-percentile |  | 36 (12.7%) | 7 (20.6%) | 0.21^a^ |
| QTc_F_ *in msec* |  | 388 (SD 24) | 396 (SD 25) | 0.10^c^ |
| QTc_F_ > 95^th^-percentile |  | 19 (6.7%) | 6 (17.6%) | **0.02^a^** |

Note: BAC = Blood Alcohol Concentration, bpm = beats per minute, ECG = Electrocardiogram, IQR = Interquartile Range, n = sample size, QTc_B_ = QT interval corrected for heart rate by Bazett’s formula, QTc_F_ = QT interval corrected for heart rate by Fridericia’s formula, SD = Standard Deviation. ^a^ = Chi-square test. ^b^ = Mann-Whitney U-test. ^c^ = ANCOVA, mean adjusted for age- and sex.

| **Supplementary Table S5** Bivariate correlations | | | | | | | | |
| --- | --- | --- | --- | --- | --- | --- | --- | --- |
| ***Parameters*** | **HR** | **Adjusted**  **p value** | **QT interval** | **Adjusted**  **p value** | **QTc_B_** | **Adjusted**  **p value** | **QTc_F_** | **Adjusted**  **p value** |
| **Demographic factors** |  |  |  |  |  |  |  |  |
| *Female sex* ^b^ | -0.10 | p=0.49 | -0.03 | p=1.00 | **+0.33** | **p<0.001** | **+0.22** | **p=0.001** |
| *Age* ^s^ | -0.06 | p=1.00 | -0.01 | p=1.00 | -0.13 | p=0.27 | -0.10 | p=0.32 |
|  |  |  |  |  |  |  |  |  |
| **Intoxication characteristics** |  |  |  |  |  |  |  |  |
| *QT-prolonging medication ^b^* | +0.04 | p=1.00 | -0.00 | p=0.98 | +0.06 | p=1.00 | +0.04 | p=1.00 |
| *BAC ^a^* | -0.12 | p=0.37 | +0.06 | p=1.00 | -0.06 | p=1.00 | -0.01 | p=0.92 |
| *Positive urine drug screening ^b^* | +0.05 | p=1.00 | -0.10 | p=0.56 | -0.09 | p=1.00 | -0.12 | p=0.32 |
|  |  |  |  |  |  |  |  |  |
| **Vital parameters** |  |  |  |  |  |  |  |  |
| *Body temperature ^a^* | **+0.18** | **p=0.02** | **-0.22** | **p=0.001** | -0.06 | p=1.00 | **-0.16** | **p=0.04** |
| *Glasgow Coma Score*^a^ | **+0.21** | **p=0.02** | **-0.22** | **p=0.001** | +0.02 | p=0.79 | -0.11 | p=0.31 |
| *Heart rate ^a^* | NA | NA | **-0.79** | **p<0.001** | NA | NA | NA | NA |
| *Systolic blood pressure ^a^* | **+0.26** | **p<0.001** | **-0.33** | **p<0.001** | -0.08 | p=1.00 | **-0.24** | **p<0.001** |
|  |  |  |  |  |  |  |  |  |
| **Laboratory results** |  |  |  |  |  |  |  |  |
| *Sodium ^a^* | +0.03 | p=1.00 | -0.09 | p=0.54 | -0.08 | p=1.00 | -0.11 | p=0.35 |
| *Log10(Potassium) ^a^* | +0.03 | p=1.00 | **-0.17** | **p=0.03** | **-0.21** | **p<0.001** | **-0.25** | **p<0.001** |
| *Calcium ^a^* | +0.14 | p=0.16 | **-0.17** | **p=0.03** | -0.04 | p=1.00 | -0.12 | p=0.33 |
| *Chloride ^a^* | -0.09 | p=0.86 | +0.12 | p=0.28 | +0.06 | p=1.00 | +0.11 | p=0.39 |
| *Log10(Glucose) ^a^* | -0.02 | p=0.75 | +0.13 | p=0.22 | +0.15 | p=0.11 | **+0.18** | **p=0.02** |
| *Arterial-blood gas pH ^a^* | +0.11 | p=0.54 | -0.10 | p=0.56 | +0.04 | p=1.00 | -0.02 | p=1.00 |
| Note: BAC = Blood Alcohol Concentration, HR = Heart Rate in beats per minute, NA = Not Applicable, QTc_B_ = QT interval corrected for heart rate by Bazett’s formula, QTc_F_ = QT interval corrected for heart rate by Fridericia’s formula. ^a^ = Pearson’s correlation. ^b^ = point-biserial correlation. ^c^ = p values are adjusted with Holm-Bonferroni method, p<0.05 is considered to be statistically significant. | | | | | | | | |
